# Supplementary material for: Time arrow in published clinical studies/trials indexed in MEDLINE: a systematic analysis of retrospective vs. prospective study design, from 1960 to 2017
Source: PeerJ. 2019 Feb 1;7:e6363. doi: 10.7717/peerj.6363 (PMC6360999; doi:10.7717/peerj.6363)
Supplement: Supplemental Information 1 [file peerj-07-6363-s001.doc]

**# Systematic Review / Meta Analysis**

**Authors who performed the Search Strategy**

MMC and PV defined the search strategy, PV performed the search strategy.

**Search strategy and solutions to disagreements**

In summary the search strategy was to query The MEDLINE database by using the keywords “retrospective study” and “prospective study”; these words were assumed as reference to describe the two types of approach. For the purpose of this research, after evaluating/comparing all the possible strategies (see limitations of the study), in agreement with our reference librarian, we relied on free text search by using the “best match”, a function available on PubMed that prevents incorrect classification problems by including MESH.

For the general statistics, even if few matching records were found starting from 1913, the “publication date” of the search query was limited by starting from 1960, where at least 10 matching documents for both category were found. Since in most cases it was not possible to find all the information needed, this sample was built starting from 2004 and placed in a comprehensive database including the over-mentioned variables. For the assessment of the country of origin and topic, we randomly sampled a minimum of 5 studies per decade where the country of origin of the first author was clearly stated and the topic was assessable by the availability of a full text.

**Notes to the revised version**

Since the search strategy may affect strongly the results, our strategy has been already checked with our librarian at out University Library (see acknowledgement); the main problems we have encountered are summarized below in the following list:

*-Search PubMed vs MEDLINE, since the first includes documents non indexed for MEDLINE;*

*-Search other databases such as EMBASE that searches MEDLINE using also MESH;*

*-Search retrospective and prospective study with or without question marks with PubMed-Best Match function that includes MESH;*

*-Search retrospective and prospective study followed or not by * to include the key word “studies”;*

*-Search excluding Book Chapters;*

*-Search excluding Vet studies or not.*

All these searches have been made previously and, whether proceeding in a restrictive or inclusive manner, the results in terms of percentage in favor of retrospective studies do not change thus, excluding macroscopic incongruities (e.g. Book Chapters), we decided to support the simplest and most reproducible strategy, that is PubMed>Best Match>MESH, providing the results updated to 31 December 2017, starting from 1960. The number of the studies is, therefore, updated in this new revised version but, as we have verified, the proportions are almost superimposable with the previous one. Finally, the prevalence of retrospectives was also verified in EMBASE. For the reviewer we report two “screen shot”.

**Rationale and contribution**

1 the rationale of the study was to assess the prevalence of retrospective vs prospective study design in clinical studies/trials published since 1960 to 2017 and available in the MEDLINE database and to establish if this prevalence has to do with the income of the country of origin of the study (first author) and with the topic of the study.

2 as far as we know, no previous studies has focused these topics over a time interval of 57 yrs; we believe that our study provides nontrivial informations on how we do research.
